# Successful Rescue Transaortic Valve Replacement Using Edwards Sapien 3 Following Failed Evolut R Implantation in a Degenerated Surgical Bioprosthesis: A Case Report

**DOI:** 10.7759/cureus.54318

**Published:** 2024-02-16

**Authors:** Victor H Molina-Lopez, Eduardo Partida-Rodriguez, Jaime Rivera-Babilonia, Luis Rodriguez-Ospina

**Affiliations:** 1 Cardiology, VA Caribbean Healthcare System, San Juan, PRI; 2 Interventional Cardiology, VA Caribbean Healthcare System, San Juan, PRI; 3 Interventional Cardiology, Hospital Pavia, San Juan, PRI

**Keywords:** bioprosthetic valve fracture, valve-in-valve-in-valve, valve-in-valve, aortic regurgitation index, prosthetic valve insufficiency, transcatheter heart valve, transcatheter aortic valve replacement

## Abstract

This study examines a complex scenario of structural valve degeneration (SVD) in a high surgical-risk patient with a previously implanted 25 mm Carpentier-Edwards (CE) Perimount Magna Ease 3300 (Irvine, CA: Edwards Lifesciences) surgical bioprosthetic valve (SAV), the patient presented with both paravalvular leak (PVL) and central prosthetic valve insufficiency (PVI). The patient was considered for a transaortic valve-in-valve (ViV) intervention with a self-expanding 29 mm Evolut R valve (Minneapolis, MN: Medtronic). The case describes a ViV intervention complicated by the malpositioning of the Evolut R valve secondary to micro-dislodgement into the left ventricular outflow tract (LVOT) after deployment and subsequent migration into the LVOT during an attempted bioprosthetic valve fracture (BVF) of the SAV that aimed to decrease transvalvular gradients. The resulting acute severe PVL resulted in significant hemodynamic deterioration, necessitating emergent intervention by implanting a balloon-expandable 26 mm Edwards SAPIEN 3 valve (Irvine, CA: Edwards Lifesciences), effectively averting the need for a surgical valve explant. This study illuminates the intricacies and emergency management strategies in transcatheter aortic valve replacement (TAVR) procedures, particularly in high-risk patients with SVD, and offers critical insights into the challenges and solutions in ViV implantations.

## Introduction

The advancement of transaortic valve-in-valve (ViV) procedures represents a significant milestone in managing structural valve degeneration (SVD), especially for patients with elevated surgical risks. This case explores a complex instance of SVD and valve dehiscence in a patient with a previously implanted 25 mm Carpentier-Edwards Perimount Magna Ease 3300 (Irvine, CA: Edwards Lifesciences) surgical bioprosthetic valve, characterized by paravalvular leak (PVL) and transvalvular prosthetic valve insufficiency (PVI) due to leaflet structural deterioration. Precision in positioning and deploying transcatheter heart valves (THV) is critical in these procedures, necessitating an in-depth understanding of valve structure and meticulous fluoroscopic guidance [1,2]. This case highlights the unique challenges encountered in patients with PVI and PVL undergoing transcatheter aortic valve in surgical aortic valve (TAV-in-SAV) interventions. It illustrates the intricate decision-making and strategic planning required to manage SVD and aortic insufficiency (AI) in high-risk patients. This report underscores the importance of individualized approaches in TAV-in-SAV procedures to ensure optimal outcomes and sheds light on the complexities and emergency strategies employed during these complex interventions.

## Case presentation

An 86-year-old male patient with recurrent hospitalizations due to heart failure and angina was assessed for the treatment of SVD presenting as aortic PVI and PVL. Six years earlier, the patient had undergone surgical aortic valve replacement (SAVR) for severe AI without aortic root enlargement, employing a 25 mm Carpentier-Edwards Perimount Magna Ease 3300 (Irvine, CA: Edwards Lifesciences) bioprosthetic valve. Concurrently, he received coronary artery bypass grafting (CABG) to address three-vessel coronary artery disease. Over the years, he experienced progressive worsening exertional angina and dyspnea, aligning with a functional classification of Class IV as per the New York Heart Association (NYHA) Heart Failure Criteria.

The echocardiographic evaluation demonstrated severe PVI due to SVD with a transvalvular insufficiency jet from leaflet degeneration and a large area of PVL due to ring dehiscence of 25% circumferential area. Physical examination was remarkable for widened pulse pressure and a diastolic murmur. The 12-lead electrocardiogram (ECG) had normal sinus rhythm. Coronary and bypass angiography was remarkable for patent bypass grafts and fully revascularized coronary anatomy. Past medical history was remarkable for recurrent admissions due to congestive heart failure with a mildly reduced left ventricular ejection fraction (LVEF) of 40-45% and global hypokinesia. The patient's history also included paroxysmal atrial fibrillation with an atrial fibrillation CHA_2_DS_2_-VASc score for stroke risk of 7 and chronic anticoagulation with apixaban, type II diabetes mellitus with diabetic nephropathy, and impaired renal function consistent with chronic kidney disease stage 3b. Given his high operative risk score (Society of Thoracic Surgeons {STS} score of 9%), he was referred for TAV-in-SAV intervention.

Computerized tomography (CT)-derived measurements were performed for procedural planning and valve sizing. The Carpentier-Edwards Magna Ease is a fracturable, stented, supra-annular aortic valve bovine bioprosthesis. The Evolut R valve (Minneapolis, MN: Medtronic) was selected due to the patient's history of ischemic heart disease, considering the valve is a self-expanding THV with a supra-annular design to optimize hemodynamics and minimize gradients. It also has an extended skirt that could provide consistent radial force and allow for optimized oversizing if SAV fracture was required.

**Figure 1:**
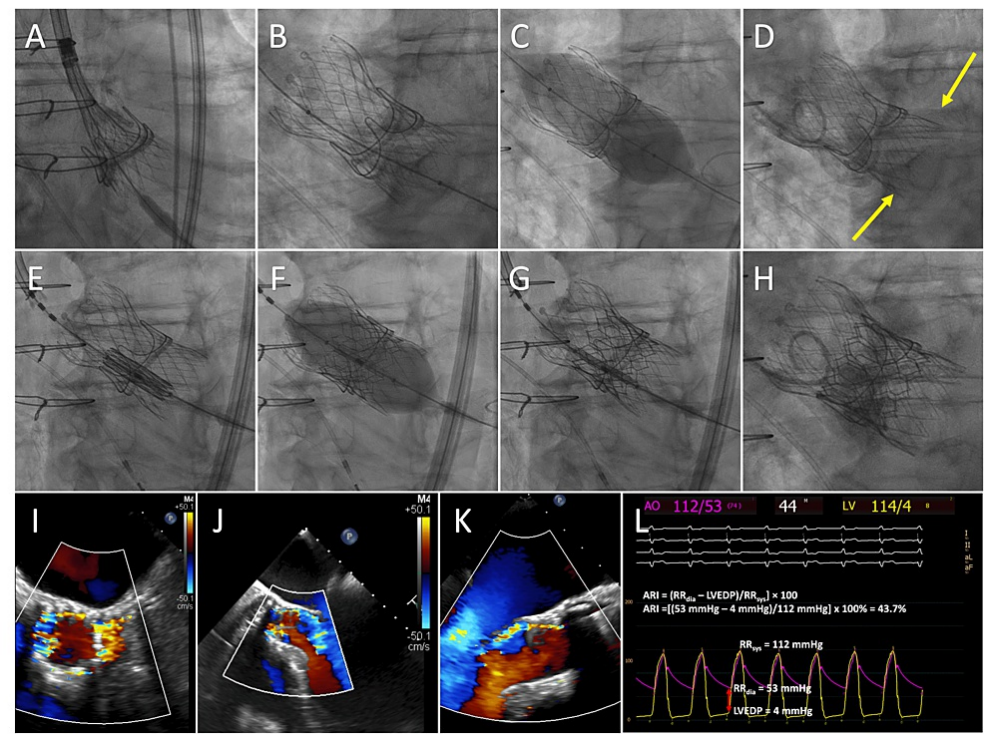
Intraprocedural TEE and fluoroscopic guidance for planned TAV-in-SAV with salvage S3-in-Evolut for treatment of severe PVL. (A) The prosthesis during the last phase of valve deployment, immediately before it is fully released. The implantation depth is measured from the hypothetical aortic annular plane to the interventricular end of the THV. Distance evaluations are made in relation to both the LCC and the NCC. (B) Micro-dislodgement of the THV occurred in the direction of the LVOT by 3 mm in relation to the LCC and 3 mm to the NCC, respectively. (C) The Magna Ease valve was cracked with a 26 mm non-compliant balloon. (D) LVOT migration up to 17 mm of the Evolut occurred after BVF with consequent acute severe PVL (yellow arrows). (I-K) TEE revealed severe supra-skirt PVL. (E) A 26 mm S3 valve was positioned with the marker on the Magna Ease stent border (F and G) and deployed, (H) successfully sealing the PVL with only trace residual PVL (L) and favorable transvalvular hemodynamics. TEE: transesophageal echocardiography; TAV-in-SAV: transcatheter aortic valve in surgical aortic valve; S3: SAPIEN 3 balloon expandable valve; Evolut: Evolut R self-expanding valve; PVL: perivalvular leak; THV: transcatheter heart valve; LCC: left coronary cusp; NCC: non-coronary cusp; LVOT: left ventricular outflow tract; BVF: bioprosthetic valve fracture; LVEDP: left-ventricular end-diastolic blood pressure; RRdia: end-diastolic blood pressure in the aorta; RRsys: systolic blood pressure in the aorta

The procedure was performed under conscious sedation by an anesthesia practitioner. A 5 Fr pigtail catheter was advanced to the aortic root via the left femoral artery. A 4 Fr balloon flotation temporary pacing wire was placed via the right internal jugular vein. The right common femoral approach was selected and pre-closed with the standard Perclose (Redwood City, California: Abbott Cardiovascular) technique. The valve was crossed with an Amplatz L1 (Miami, FL: Cordis Corp.) catheter, a hand-carved J-tip Amplatz SuperStiff guidewire (Miami, FL: Boston Scientific Corp.) was coiled in the LV, and an 18 Fr Cook sheath was introduced over the wire. A 29 mm Evolut R was inserted and positioned using fluoroscopic guidance without prior valvular pre-dilation while pacing at 150 beats/minute to mitigate the impact of THV movement. Standard slow deployment was performed with an implant depth goal of 4-6 mm below the sewing ring marker under fluoroscopic guidance. Multiple attempts to reposition the valve were made, eventually achieving the desired implant depth (Figure 1, panel A) but micro-dislodgement into the left ventricular outflow tract (LVOT) after full deployment was noted (Figure 1, panel B). There was trace PVL by angiography. However, the transvalvular mean gradient was 18 mmHg. Using a 26 mm True Dilatation non-compliant balloon (Murray Hill, NJ: Bard), the Magna Ease valve was cracked with good expansion (Figure 1, panel C). However, the THV valve further migrated into the LVOT with progressive dyspnea and hypoxemic respiratory failure, requiring endotracheal intubation and mechanical ventilation.

The resulting deep implantation of the 29 mm Evolut R prosthesis as a consequence of both micro-dislodgement and LVOT migration after BVF led to severe PVL with a marked widening of the pulse pressure on invasive hemodynamics and an aortic regurgitation index (ARI) of 18%. Intraprocedural transesophageal echocardiogram (TEE) revealed massive circumferential supra-skirt PVL (Figure 1, panels I-K). Snaring the Evolut valve was considered but deferred due to the risk of aortic dissection or possible recurrence of valve migration.

The subsequent insertion of a third THV, this time employing a balloon-expandable 26 mm Edwards SAPIEN 3 (Irvine, CA: Edwards Lifesciences) with a slow deployment technique led to successful anchoring on the Evolut frame, positioned with the marker on the Magna Ease stent border at 15% depth (Figure 1, panel E). Deployment successfully sealed the annulus with angiography revealing only trace residual PVL (Figure 1, panel H). The ARI notably improved to 43.7%, indicating a successful sealing of the PVL (Figure 1, panel L). The transvalvular mean gradient after the valve-in-valve-in-vale (ViViV) procedure with an S3-in-Evolut-in-Magna Ease intervention was 8 mmHg. Final aortography and hemodynamic monitoring confirmed trace PVL. A post-procedure transthoracic echocardiogram (TTE) showed normal functioning of the ViViV S3 with trace PVL and no patient-prosthetic mismatch (PPM). Post-procedure acute left bundle branch block (LBBB) was noted. However, resolved before discharge from the hospital. The patient was followed closely for the next three years and remained without complications from the intervention or early SVD, passing away three years later from non-cardiovascular etiology.

## Discussion

In the context of TAV-in-SAV interventions, achieving accurate positioning and deployment of a THV is paramount. This process demands meticulous procedural planning, precise recognition of the stented valve structure, and careful fluoroscopic guidance to ensure successful outcomes. When considering transcatheter aortic valve replacement (TAVR) for cases of native aortic valve insufficiency, practitioners face distinct challenges. The absence of calcification in the leaflets or annulus can complicate secure valve anchorage, elevating the risks of valve embolization or migration. Additionally, such cases may present an increased likelihood of PVL, along with potential conduction system abnormalities [3,4]. Nevertheless, in TAV-in-SAV scenarios, the existing stent frame can provide a reliable anchoring mechanism. Aortic insufficiency often leads to increased stroke volumes. In instances of more than mild insufficiency through a bioprosthetic valve, the consideration for ventricular pacing becomes an essential strategy. Furthermore, ensuring the guidewire's stable positioning and the THV's coaxial implantation are fundamental steps to secure the valve's accurate implant. 

The Magna Ease aortic valve is a third-generation bioprosthetic valve designed from the Perimount and Magna valve designs. SVD has been observed in 28.7% of patients at an average of 3.9 years post-implantation. Moderate or severe prosthetic regurgitation has been noted in 3.9% of patients, excluding those with post-operative endocarditis. About 0.3% undergo re-operation or re-intervention for SVD, typically at a median of 2.4 years [5]. Bioprosthetic SAV failure can result from calcification, wear and tear, thrombosis, pannus formation, or endocarditis. The most common cause of bioprosthetic valve failure is leaflet tissue deterioration. Calcific deposits usually develop where leaflet flexion and stress are greatest, usually at the basal and commissural attachment points. About 75% of patients with leaflet tears and calcification suffer from aortic regurgitation [6].

The Carpentier-Edwards Magna Ease 3300 has an intraannular stent design, with leaflets sutured inside the stent. The sewing ring is above the lowest part of the stent, which can be used for fluoroscopic guidance. It is supported by a cobalt-nickel (Elgiloy) wire form stent with an Elgiloy and polyester ring and stent posts. It is designed for supra-annular implant positioning. The sewing ring's position offers the smallest diameter within an implanted SAV and serves as a reference point or a "neo-annulus" for anchoring the THV securely. In stented bioprosthesis, the sewing ring is the most rigid structure and provides the anchor for THV implantation. An Evolut R valve should be implanted approximately 4 mm below the lower stent border for best outcomes. Meanwhile, a SAPIEN S 3 valve should be implanted 15% below the marker [7]. Careful initial placement of the Evolut is crucial, as repositioning the THV becomes highly challenging or may not be feasible once it has established firm contact with the surgical valve's frame.

The Magna Ease valve is fracturable, and implantation strategies considering BVF should consider device specifications for correct non-compliant balloon sizing. A significant complication associated with valve-in-valve therapy is the "Russian Doll" effect [6,7]. This arises when a THV is inserted within an index SAV, rendering its expansion and operational capacity contingent upon the index valve's internal diameter (ID). Balloon pressure from within can counteract this effect by expanding the “neo annulus” and increasing the index valve's true internal diameter (TID). The size of the balloon should be at least equal to the stent size. When a BVF strategy is considered, valve cracking can be done before or after the ViV implant [8,9]. However, the preferred strategy is to implant the new THV and then perform valve cracking with a dilation balloon [7,8,10,11]. For the index valve, in this case, a True Dilatation balloon size of 26 mm was appropriate as guided by pre-procedural CT measurements. After BVF, the subsequent required THV size may be larger to properly seal the annulus.

The ideal size selection of an Evolut R valve for a ViV strategy in cases of an index 25 mm CE Magna Ease valve should consider the size of the sinus of Valsalva and consider the Evolut implant depth at approximately 4 mm from the lowest visible margin of the Magna Ease stent by fluoroscopy [7]. The Evolut 29 mm has an annulus diameter of 23-26 mm. This case's initial planned implant depth was 4-6 mm due to the lower right coronary artery (RCA) coronary ostia. However, the undesired THV migration into the LVOT required a change in strategy for the second THV implant. This requires positioning the S3 within the Evolut at the most optimal location to avoid compromising coronary access, coronary flow, or leaflet function. In the in vitro study by Akodad et al. in 2022, a 26 mm S3 implanted in a 29 mm Evolut R valve with S3 outflow positioned at node 6 of the Evolut R has the lowest leaflet overhang (3%), best indexed effective orifice area (EOAi) (2.3 cm^2^), lowest mean gradient (8.9 mmHg), and lowest total regurgitation fraction (15%) [12]. Also, a high S3 implantation in node 6 is associated with a taller neo-skirt. However, in the setting of a migrated Evolut R into the LVOT, it does not necessarily translate to compromised coronary access if the S3 is appropriately positioned relative to the neo-annulus from the Magna Ease SAV. The ViV estimated diameter for sizing an S3 implant at node 5 of the Evolut R is 26.5 mm. The likelihood of S3 displacement occurring within the Evolut R framework is estimated to be minimal across various depths of implantation [12]. An implantation technique for the S3 involving higher final valve depth might help prevent post-TAVI permanent pacemaker implantation (PPMI) without affecting the risk of PVL [13]. This increase in the PPMI rate can be avoided by intending an aortic stent extension of the S3 greater than 70% [14].

Decisions to treat PVL ad hoc would depend on the severity and mechanisms of PVL to prevent early SVD and PPM and improve procedural outcomes. The simultaneous measurement of left ventricular end-diastolic pressure (LVEDP), diastolic blood pressure (DBP), and systolic blood pressure (SBP) in the aorta can be used to assess the severity of PVL. This can be done to calculate the ARI using the following formula: ({DBP - LVEDP}/SBP) × 100 [15]. In a case series by Sinning et al. in 2012, patients with an ARI greater than 31.7±10.4 exhibited no significant PVL, whereas those with an ARI of 19.6±7.6 had moderate PVL, and those with an ARI of 7.6±2.6 experienced severe PVL. An ARI below 25 was linked to a higher one-year mortality risk compared to those with an ARI above 25 (46% vs 16.7%) [15]. Thus, for patients exhibiting more-than-mild PVL or an ARI <25, it is advisable to evaluate with echocardiography (ideally TEE), and establish the underlying reasons and mechanisms of the PVL [15,16].

PVL jets secondary to THV malposition can occur when a prosthesis is placed too low or too high relative to the native annulus. In cases of low implantation, the THV is deployed deeper than its tissue skirt's height, leading to a PVL jet above the skirt (supra-skirt) from the aortic stent portion into the paravalvular space and LVOT [16]. Conversely, with high implantation, the THV is positioned partly above the native annulus, leading to the PVL jet to flow from the paravalvular space through the prosthesis's irregular inflow edge into the LVOT (infra-skirt) [16]. Following interventions aimed at addressing the clinically significant PVL, the extent of AI should be reassessed using imaging techniques and the ARI to gauge the effectiveness of the corrective measures [16].

It is important to note that ViV therapy presents a theoretically increased risk of thrombosis. In ViV implants, bioprosthetic leaflets form a cylindrical enclosure for the THV, impeding direct blood flow over the leaflets and leading to stasis, particularly in more complex ViViV procedures. This necessitates consideration of anticoagulation to avert thrombosis and early SVD. Using a self-expanding prosthesis at a higher implant position is linked to decreased stasis in the neo-sinus flow, where the washout of the native sinus and coronary flow relies on the cardiac output [17]. Also, stasis in the neo-sinus flow can be determined by the expansion at which the THV is implanted [18]. Studies such as the Valve-in-Valve International Data (VIVID) Registry have shown a high incidence of valve thrombosis in as high as 7.6% of cases of ViV TAVR, but this risk drops to 1.0% in patients on oral anticoagulants for other conditions, compared to 11.3% in those not on anticoagulants [19]. Current guidelines favor single antiplatelet therapy post-TAVR but leave some questions unanswered for the ViViV subgroup [20]. The choice of therapy post-ViV TAVR remains at the clinician's discretion, pending further research to establish a standardized treatment protocol for these patients.

## Conclusions

This study exemplifies the complexities and adaptive strategies essential in TAV-in-SAV procedures, particularly in managing SVD in high-risk patients. The intricate interplay of procedural challenges, including the micro-dislodgement and migration of the Evolut R valve into the LVOT, resulted in severe PVL and acute symptomatic PVL. The strategic insertion of the S3 valve within the existing Evolut R and Magna Ease framework proved to be a decisive and effective "rescue" maneuver. This intervention not only rectified the immediate complications but also significantly enhanced the valve's overall function and hemodynamic performance. The resolution of PVL and improvement in the ARI underscore the necessity of precision and adaptability in interventional cardiology. Moreover, this case underscores the viability and efficacy of the S3-in-Evolut implantation strategy in complex ViV scenarios, reinforcing the need for individualized treatment approaches in TAV-in-SAV interventions. The successful outcome of this case contributes valuable insights into the evolving landscape of transcatheter heart valve therapies and highlights the importance of continuous innovation and flexibility in the face of procedural unpredictability.
